# Natural Dietary and Herbal Products in Anti-Obesity Treatment

**DOI:** 10.3390/molecules21101351

**Published:** 2016-10-11

**Authors:** Nan-Nong Sun, Tsung-Yen Wu, Chi-Fai Chau

**Affiliations:** 1 Department of Food Science and Biotechnology, National Chung Hsing University, Taichung 40227, Taiwan; b506096053@gmail.com (N.-N.S.); binging0168@icloud.com (T.-Y.W.); 2 Agricultural Biotechnology Center, National Chung Hsing University, Taichung 40227, Taiwan

**Keywords:** anti-obesity, weight loss, phytochemicals, dietary supplements, mechanism

## Abstract

The prevalence of overweight and obesity is on the rise around the world. Common comorbidities associated with obesity, particularly diabetes, hypertension, and heart disease have an impact on social and financial systems. Appropriate lifestyle and behavior interventions are still the crucial cornerstone to weight loss success, but maintaining such a healthy lifestyle is extremely challenging. Abundant natural materials have been explored for their obesity treatment potential and widely used to promote the development of anti-obesity products. The weight loss segment is one of the major contributors to the overall revenue of the dietary supplements market. In this review, the anti-obesity effects of different dietary or herbal products, and their active ingredients and mechanisms of action against obesity will be discussed.

## 1. Introduction

Obesity and overweight are major contributors to the global burden of chronic diseases and complications, including cardiovascular diseases, diabetes, and cancer. In 2014, it was reported by World Health Organization (WHO) that more than 1.9 billion adults were overweight, and of these over 600 million were obese. Once mainly a concern for higher income countries, overweight and obesity are also on the rise in low and middle income countries, especially in urban areas [1].

There are many different options for obesity treatments, including dietary control, exercise, life-style changes, prescription weight-loss medications, and weight-loss surgeries [2]. According to the “Pharmacological Management of Obesity: An Endocrine Society Clinical Practice (ESCP) Guideline”, recommended by the National Heart, Lung, and Blood Institute [3], the most ideal treatment modality for weight loss should be appropriate dietary and lifestyle changes plus moderate-intensity exercise. However, many epidemic and clinical studies have shown that it is a great challenge to maintain long-term lifestyle modification [4]. Obese patients are easily frustrated during the painful and seemingly endless lifestyle changing progression. Providing ways to accelerate weight loss could inspire obese patient’s confidence to accomplish their goals and give them more motivation to change their lifestyle behavior.

In 2007, the Nutrition Business Journal showed that the aggregate market value of dietary supplement reached nearly 20 billion dollars in sales for the USA. It was estimated that there was at least a 700 million dollars market for weight loss products alone in 2008 [5]. By the end of 2015, the global dietary supplements market is estimated to reach nearly 123 billion dollars. In terms of the market segmentation by application, the weight loss segment is one of the major contributors to the overall revenue of the dietary supplements market. The development of the weight loss segment is anticipated to register a compound annual growth rate of 7.4% over the forecast period 2015–2025 [6]. Thus, natural supplements products primarily helping consumers to fight the battle against obesity have been widely explored. A variety of natural plants (e.g., herbs, fruits, and vegetables), functional fatty acids (e.g., polyunsaturated fatty acids and conjugated fatty acids), and other natural dietary compounds have been used in different anti-obesity products. Natural plant products are expected to be potential ingredients for the development of nature-sourced anti-obesity products in the weight loss segment due to rising consumer health awareness. In this study, the anti-obesity effects of different dietary or herbal products, and their active ingredients and mechanisms of action against obesity will be discussed.

## 2. Development of Anti-Obesity Drugs

Anti-obesity drugs have been studied profoundly for decades. The need for adjunctive therapies for weight loss has accelerated the progress in the pharmaceutical industry worldwide. In USA, four FDA-approved oral weight loss drugs have been developed, including orlistat (marketed as Alli and Xenical), Contrave (naltrexone hydrochloride and bupropion hydrochloride extended-release tablets), Belviq (lorcaserin hydrochloride), and Qsymia (phentermine and topiramate extended-release). These clinical medications manipulate body weight by increasing energy expenditure, suppressing appetite, or inhibiting pancreatic lipase to decrease lipid absorption in the intestine [7,8].

Weight loss drugs may appear to be a solution to obesity. However, possible side effects or adverse drug reactions are always a big public health concern and also a major barrier to the development of new drug products. For example, in 1997, two weight loss drugs, fenfluramine (part of the popular fen-phen) and dexfenfluramine (Redux), were withdrawn from the market because of their possible detrimental effects to heart valves. In 2010, sibutramine (Meridia) was also withdrawn due to an increased risk of heart attacks and strokes. In the same year, in response to the occasional reports of severe liver injury with the use of weight loss drug “Xenical”, FDA approved a revised drug label in which safety information about its potential side-effect was noticed.

As mentioned in the ESCP Guideline, pharmacological obesity treatment should be intended only for patients with BMI > 30 or BMI ≥ 27 with comorbidity [3]. Considering the potential side effects, anti-obesity drugs should be prescribed for obesity only if the benefits of the treatment outweigh the risks.

In face of the adverse side effects of synthetic drugs, natural products are now preferably used due to their effectiveness in managing overweight and many other chronic disorders. For example, traditional herbal medicines with a long history of use along with other natural substances might suppress appetite and promote weight loss. It was in general believed that these natural materials could be relatively more economical with little to no toxic side effects when compared with the synthetic ones [2].

## 3. Anti-Obesity Mechanisms of Natural Dietary or Herbal Products

### 3.1. Increase in Energy Expenditure

Excessive adiposity can primarily be attributed to an imbalance in energy homeostasis, in which the consequences of excessive food intake are not balanced by increased energy expenditure [9]. That is the reason why increasing energy expenditures to build a negative energy balance is crucial to weight management. Energy expenditure can be briefly classified into three categories: (1) physical activity; (2) obligatory energy expenditure; and (3) adaptive thermogenesis. Most anti-obesity products usually regulate body weight through an increase of obligatory energy expenditure. In the human body, the function of brown adipose tissue is to transfer energy from food into heat. Uncoupling protein 1 (also known as thermogenin) in brown adipose tissue plays a key role in thermogenic effect. Therefore, a material capable of upregulating uncoupling protein 1 gene expression could be a potential strategy for achieving an anti-obesity effect by increasing energy expenditure [10].

### 3.2. Appetite Suppressant Effect

There has been a rise in satiety-enhancing products on sale in the food supplement marketplace. The biological mechanisms of appetite and satiety are regulated by a complex interaction of neurological and hormonal signals. Many studies have revealed that some food ingredients could provide satiety enhancing effects and be beneficial for weight control [11]. The mechanism underlying the enhanced satiety includes an increase of noradrenaline level and subsequent activation of sympathetic nervous system activity, leading to an increase in satiety and energy expenditure, suppression of hunger, and also elevation in fat oxidation [12].

A line of evidence indicates that neural signal peptides like serotonin, histamine, dopamine, and their associated receptor activities are associated with satiety regulation. These neural signal peptides and their receptors could be potential target areas for the development of supplement products that treat obesity through energy intake reduction by increasing satiety [13].

### 3.3. Lipase Inhibitory Effect

One of the promising strategies for treating obesity is to interfere with fat absorption along the gastrointestinal tract directly (without altering the central nervous system). There has been long-standing interest in discovering and developing inhibitors for nutrient digestion and absorption. The underlying concept is that for any dietary fat being absorbed in human intestine, the fat should be broken down enzymatically by the action of pancreatic lipase. Pancreatic lipase activity is therefore widely considered as one of the most important indicators for the determination of the anti-obesity potential of natural products [14].

As a key enzyme in dietary triglyceride absorption, pancreatic lipase hydrolyzes triglyceride to monoglyceride and fatty acids. Some substances have been confirmed to have capabilities to interact directly with intestinal lipases themselves. One well known example is tetrahydrolipstatin (orlistat), a derivative of the naturally-occurring lipase inhibitor isolated from *Streptomyces toxytricini* [15]. It is a synthetic drug designed to act through a covalent bond to the active site serine of pancreatic lipase to block the absorption of dietary fat [16,17].

### 3.4. Regulatory Effect on Adipocyte Differentiation

Adipocytes play a central role in the maintenance of lipid homeostasis and energy balance. They have a particular large capacity to store triglycerides as well as to release free fatty acids in response to changing energy demands. As adipocyte tissue growth is associated with both the hyperplasia and hypertrophy of adipocytes, this has led to the development of natural products in anti-obesity therapy that specifically target adipogenesis inhibition. Some research has also suggested that adipocyte differentiation could be inhibited by the blockade of several transcription factors such as C/EBPβ (CCAAT/enhancer binding protein beta) and PPARγ (peroxisome proliferator-activated receptor gamma) [18]. A study on the inhibitory activity of *Sibiraea angustata* extracts on adipocyte differentiation has also revealed that the expression of both C/EBPβ and PPARγ were significantly inhibited leading to a decrease in the lipid content of adipocyte cells [19].

### 3.5. Regulatory Effect on Lipid Metabolism

An increase in the rate of lipolysis stimulates triglyceride hydrolysis and hence diminishes fat storage and combats obesity. The key points to enhance lipolysis have been discussed in many studies. For instance, activation of β-adrenergic receptor initiatively triggered lipolysis in white adipocytes and non-shivering thermogenesis in brown fat [20]. An activation of adenosine monophosphate-activated protein kinase (AMPK) leading to the increase of fatty acid oxidation and glucose transport in skeletal muscle was another example [21,22]. Hence, transcription factors that can simulate lipolysis become an important feature in the development of anti-obesity products. It has been shown that flavonoids, specifically flavonols (e.g., quercetin), could activate lipoxygenase and attenuate adipogenesis through the up-regulation of the AMPK pathway. Additionally, quercetin activates the apoptotic pathway in mature adipocytes through suppression of phosphorylation effect of signal-regulated kinases 1 and 2 (ERK1/2) and c-Jun N-terminal kinase (JNK), both belonging to subfamilies of mitogen-activated protein kinase (MAPK) [23].

## 4. Anti-Obesity Products from Natural Resources

The growing threat of obesity to global health has encouraged scientists and researchers to put more effort into finding an efficient anti-obesity ingredient. Numerous potential materials from natural sources have been investigated along with their active ingredients. These natural materials are mostly derived from plants, including fruits, vegetables, grains, and herbs. The biological benefits of these natural materials are basically contributed by the presence of an abundant amount of phytochemicals, fibers, and unsaturated fatty acids [24]. Some selected natural resources along with their anti-obesity effects and active ingredients are summarized in Table 1, and some notable examples [25,26,27,28,29,30,31,32,33,34,35,36] will be further discussed.

The anti-obesity products in the market can be classified into three categories: (1) food ingredients; (2) herbal ingredients; and (3) other functional supplements. Developing functional products from what people usually consume in their daily life is probably the most popular segment in the functional supplement industry. Products made from fruits (citrus, and berries), grains (soybean), vegetables, or beverage drinks (tea leaves) are relatively safer and more acceptable to consumers. Nowadays, traditional Chinese medical practitioners use herbal remedies that usually are combinations of different herbs, such as turmeric (*Curcuma longa*) and mulberry leaf (*Morus alba*), to treat obese patients. Recently, herbal therapies are not only popular in Asia, but also more and more common in the Western world. This is the reason why herbal materials could be another major category of anti-obesity products [37]. Some other materials, e.g., probiotic and calcium supplements, have also been proven to confer anti-obesity effects.

Previous studies have explored the potential health benefits of fruits. The examples of potential health benefits include anticancer, anti-inflammatory, and anti-obesity effects [38]. Citrus fruit is one of the major categories used for the exploration and exploitation in new anti-obesity products. Phytochemicals including triterpenoids, flavonoids, and alkaloids are candidate ingredients that are found to be abundant in both the peel and pulp of citrus fruits. Cell and animal studies have shown the anti-obesity effects of citrus fruit extracts that help lower body weight gain and white adipose tissue weight. Leptin, which is a key hormone produced by adipocytes and functioning in the regulation of food intake and energy expenditure, was found to be reduced by the intake of citrus fruits. This change in hormonal activity is desirable for the development of citrus-based anti-obesity product. In citrus fruits, methoxylated flavones and flavanone glycosides are the major bioactive flavonoid compounds capable of changing plasma leptin levels.

Anti-obesity products derived from green tea (*Camellia sinensis*) leaves are also popular in functional food market. The major bioactive ingredients constituent in green tea, accounting for up to 35% of the dry weight, is polyphenols. They may include flavonols, flavones, and flavan-3-ols (catechins). A number of clinical trials have revealed the beneficial effects of catechins (270 to 1200 mg/day), e.g., reduced body weight, lowered serum leptin levels, and reduced absorption of fatty acid. Another bioactive constituent in tea leaves is caffeine, which influences somatic nervous system activity and acts synergistically with catechins to increase energy expenditure and fat oxidation. Apart from green tea, other herbal tea materials such as maté tea (*Ilex paraguariensis*), rooibos (*Aspalathus linearis*), and honeybush (*Cyclopia intermedia*) have also been studied for their roles in obesity prevention and lipid metabolism [39].

Owing to the wide array of natural products that have potential anti-obesity effects, it is difficult to elaborate on all of them in one study. Scientists are devoted to finding more potential materials. In the world of published studies regarding functional supplements, there seems to be a positive bias. It is worth noting that the doses being studied in many experiments are usually much higher than the actual doses being used in commercial products [5].

## 5. Functional Ingredients in Natural Anti-Obesity Products

### 5.1. Phytochemicals

It is well known that the consumption of phytochemicals can have a major contribution to biological effects. The mechanisms of action of phytochemicals include: (1) inhibition of proliferation of precursor cells; (2) increase of apoptosis effect; (3) inhibition of pancreatic lipase activity; and (4) increase in energy expenditure [40]. Some selected phytochemicals that provide anti-obesity effects are briefly described below. A summary of different phytochemicals with anti-obesity effects IS presented in Table 2 [23,41,42,43,44,45,46,47,48,49,50,51,52,53,54].

Polyphenols are functional compounds that have anti-carcinogenic, anti-oxidant, anti-bacterial, and anti-viral activities [55]. In the past two decades, polyphenols have also been reported to have beneficial effects against obesity. For example, dietary polyphenols could regulate adipocyte metabolism to inhibit the growth of adipose tissue [56]. Phenolic acids, flavonoids, and stilbenes are the common polyphenols being used in the development of different natural weight management products.

Naturally occurring phenolic acids comprise two classes: hydroxycinnamic acids and hydroxybenzoic acids. Hydroxycinnamic acid derivatives, i.e., coumaric acid, caffeic acid, and ferulic acid, are present mainly in the form of simple esters with glucose or quinic acid. The most commonly occurring acid derivative is chlorogenic acid. In the investigations of dietary phenolic acids on mouse pre-adipocytes, chlorogenic and coumaric acids effectively exerted inhibitory effects on cell growth and enhanced apoptosis. A recent study evaluating the effects of ferulic acid administration on lipid metabolism of mice has found that it could suppress the weight gain due to inhibition of fatty acid biosynthesis [57].

Flavonoids are abundantly present in nature. They have been proven to have the positive effects on anti-obesity. Six subgroups of flavonoids are summarized in Table 2. These include flavonols, flavanones, isoflavonoids, flavones, flavans-3-ol, and anthocyanins. Flavonoids can modulate numbers of cell-signaling pathways to affect carbohydrate digestion, fat deposition, release rate of insulin, and glucose uptake in insulin-responsive tissues.

Phytosterols, which encompass plant-derived sterols and stanols, are compounds structurally similar to cholesterol. They occur in high concentrations in vegetable oils such as corn, soybean, and sunflower oil [58]. Plant stanols and sterols have been proved capable of blocking the absorption of intestinal fatty acid and reducing body weight gain in animal tests [59].

Alkaloids are generally defined as basic (alkali-like), nitrogen-containing organic constituents commonly found in plants. Alkaloids such as capsaicin and caffeine have been reported to be able to significantly increase energy expenditure, reduce appetite, and inhibit both adipocyte differentiation and pancreatic lipase. Most of these alkaloids are α-adrenergic agonists although some of them have β-adrenergic agonist properties.

### 5.2. Polyunsaturated Fatty Acids (PUFAs)

The potential anti-obesity effects of PUFAs might be explained by their performance in the following aspects: a balance between energy intake and energy expenditure, lipid metabolism, status of adipocytes, and neuroendocrine system [60]. It has been demonstrated that PUFAs could reduce the activity of the key enzymes responsible for lipid synthesis, such as fatty acid synthase and stearoyl-CoA desaturase-1 [61]. Thus, they might avoid free fatty acids entering adipocytes for lipogenesis and also improve lipid oxidation and thermogenesis [62]. Despite large amounts of investigations on the anti-obesity effect of PUFAs, the precise molecular mechanisms underlying the body-fat lowering effects of PUFAs remain largely unknown.

### 5.3. Dietary Fiber

Some anti-obesity products are rich in different types of dietary fiber, such as pectin, gum, cellulose, and soluble dietary fiber. In 1970, Heaton summarized the anti-obesity functions of dietary fiber. He has found that dietary fiber could act like a physiologic obstacle to lower energy intake by three mechanisms: (1) displacement of other nutrients in the diet by dietary fiber; (2) providing satiety and reducing appetite; and (3) inhibiting food absorption in small intestine [63].

Furthermore, recent studies have revealed that viscosity and fermentability are two important physicochemical properties that are closely related to the beneficial physiological effects of dietary fiber [64]. Many soluble dietary fibers (e.g., gums, pectins, and β-glucans) become thicken while mixing with liquids. Viscosity is a major contributor to physiological effects in the small intestine. An increase in viscosity may present a barrier to slow gastric emptying and delay nutrient absorption. Dietary fiber could also be a fermentable substrate for the colon microbiota, supporting an increase in microbial mass increase and production of short chain fatty acids. A lot of prospective studies have indicated that long-term consumption of fiber-rich diet has a negative correlation with body weight gain [65]. It has almost become a common sense that dietary consumption is crucial in the fight against metabolic disease.

### 5.4. Protein

Many protein supplements such as whey, casein and soy protein have been sold and marketed as an anti-obese product for a long while. Going on a high-protein daily diet could help people lose weight and prevents weight gain rebound [66]. Protein is more satiating than carbohydrate, and is also associated with a greater diet-induced thermogenesis [67]. Previous studies have shown that high-protein intake may induce an increased level of plasma peptide tyrosine-tyrosine which is a key inhibitor of food intake in humans and rodents [68]. The high thermogenesis of protein may be explained by the lack of storage capacity in the body, the high ATP cost of protein synthesis, and the metabolic costs of urea synthesis. It should be noted that the potential anti-obesity effect of any protein supplements could only be exerted in a more efficient manner while being consumed with adequate exercise [69].

### 5.5. Dietary Calcium

Dietary calcium is an important factor in the maintenance of skeletal integrity, blood calcium level, and modulation of chronic diseases risks. Several authors have reported that a high intake of calcium may increase fecal fat excretion and energy expenditure [60]. The mechanism of increasing fecal fat excretion is most probably due to the formation of insoluble calcium-fatty acid soaps and/or binding of bile acids [66]. Another study has demonstrated that intracellular calcium plays a key role for regulating adipocyte metabolism. It is supposed that high dose of dietary calcium could modulate circulating calcitriol levels. It in turn decreases intracellular calcium and affects fat metabolism in human adipocytes based on the findings from different cellular studies, animal studies, epidemiological studies, and clinical trials [70]. Nevertheless, some meta-analyses have argued that positive correlation between calcium intake and weight loss might not always be seen [71].

### 5.6. Probiotics

Some studies have provided evidence to support the hypothesis that gut microbes are involved in the development of type 2 diabetes mellitus and obesity [72]. Lipopolysaccharide occurred in the cell wall of gram-negative bacteria (such as *Escherichia coli* and *Enterobacter cloaca* B29) might trigger high fat diet-induced obesity [73]. The ability of some probiotics (e.g., *Lactobacillus gasseri* SBT2055 and *Bifidobacterium breve* B-3) to decrease body weight and body fat in obese patients has been proven by several clinical studies [74,75]. Mechanisms of action underlying the anti-obesity effects of probiotics include appetite regulation, host metabolism, inhibition of lipid absorption, maintenance of intestinal homeostasis and integrity, and low-grade inflammation have been proposed [76]. Using probiotics or prebiotics in combination would be a better strategy to prevent or alleviate obesity problems.

## 6. Conclusions

Obesity is a complex, chronic disorder caused by an interaction of contributing factors, including dietary, lifestyle, genetic, and environmental factors. Appropriate lifestyle and behavior interventions are the fundamentals of weight loss success, but maintaining such a healthy lifestyle is extremely challenging. The use of some natural anti-obesity products could be considered as a supportive tool to keep obese people holding on their weight-loss goals. It is also possible that the combination of multiple natural products could confer a synergistic activity that increases their anti-obesity action on multiple targets, offering advantages over chemical treatments in terms of serious side-effects. Natural materials may give not only anti-obesity effect but also other health benefits, such as anti-diabetic and anti-hyperlipidemic activities. It is anticipated that the availability of many natural sources will provide a beneficial basis for developing novel anti-obesity products.

## Figures and Tables

**Table 1:** Summary of natural materials and ingredients with potential anti-obesity effects.

Natural Materials	Bioactive Ingredients	Experimental Details	Major Activities	References
Shiikuwasa (*Citrus depressa*)	Flavonoids	1.0%–1.5% methanolic extract; male ICR mice fed with HFD (fat: 40%, *w*/*w*) for 4 weeks	10.0% reduction in body weight; 50.9% reduction in organ WAT weight	[25]
Blueberry (*Vaccinium ashei*) and Mulberry (*Morus australis*)	Anthocyanins	mixed juice; male C57BL/6 mice fed with HFD (fat: 45%, *w*/*w*) for 12 weeks	7.30%–9.81% reduction in body weight; reduction in WAT adipocyte sizes	[26]
Soybean (*Glycine max*)	Protein isolated	30% protein isolate; male SD rat fed with HFD (fat: 25%, *w*/*w* ) for 180 days	18.9% reduction in body weight gain; 26.6% reduction in body fat; higher thermogenin expression	[27]
Coffee (*Coffea Arabica*)	Caffeoyl, quinic acids	0.5% to 1.0% aqueous extract; male C57BL/6J mice fed with HFD (fat: 30%, *w*/*w*) for 2–15 weeks	14.3% reduction in body weight; 16.2% reduction in organ WAT weight; decrease in liver fat	[28]
Green tea (*Camellia sinensis*)	Ppolysaccharides, caffeine and catechins	400 to 800 mg/kg aqueous extract; male SD rats fed with HFD (fat: 10%, egg yolk powder10%, *w*/*w*) for 6 weeks	11.3%–16.9% reduction in body weight; reduction in body fat index and WAT adipocyte sizes	[29]
Lotus leaf with taurine (*Nelumbo nucifera*)	Alkaloids, flavonoids, triterpenoids, polyphenols, steroids, glycosides, and taurine	400 mg/kg aqueous extract with taurine; male SD rats fed with HFD (fat: 20%, *w*/*w*) for 6 weeks	Reduction in body weight; reduction in WAT adipocyte sizes and number	[30]
Ginger (*Zingiber officinale*)	Gingerol, paradol, and shogoal	5% ginger powder; male albino rats fed with HFD (fat: 30%, *w*/*w*) for 5 weeks	38.6% reduction in body weight; 45.7% reduction in pancreatic lipase activity	[31]
Black wattle (*Acacia mollissima*)	Robinetinidol and fisetinidol	5.0% aqueous extract; diabetic KKAy mice fed with HFD (fat: 60%, *w*/*w*) for 7 weeks	23.2% reduction in body weight; Higher expression of energy expenditure-related genes	[32]
Chili pepper (*Capsicum annuum*)	Capsaicin	10 mg/kg b.w. capsaicin male SD rats fed with HFD (fat: 45%, *w*/*w*) for 9 weeks	8% reduction in body weight; reduction in WAT weight and adipocyte sizes	[33]
Coptis Root (*Rhizoma coptidis*)	Berberine	200 mg/kg ethanolic extracts; male C57BL/6J mice fed with HFD (fat: 16.2%, *w*/*w*) for 6 weeks	Reduction in body weight; reduction in organ WAT weight	[34]
Turmeric (*Curcuma longa*)	Curcumin	50% ethanolic extract; male SD rats fed with HFD (fat: 60%, *w*/*w*) for 12 weeks	15.9% reduction in body weight gain; 31.3% reduction in organ WAT weight	[35]
White mulberry (*Morus alba*)	Rutin, resveratrol anthocyanin, and 1-deoxynojirimycin,	combination of leaves extract (133–333 mg/kg) and fruit extract (67–167 mg/kg) male C57BL/6 mice fed with HFD (kcal: 45% from fat) for 12 weeks	53.5% reduction in body weight gain; reduction in organ WAT weight	[36]

HFD, high fat diet; b.w., body wieght; WAT, white adipose tissue; SD rats, Sprague Dawley rats.

**Table 2:** Phytochemicals with anti-obesity effects.

Groups	Active Ingredients	Structures	References
Phenolic acids	o-Coumaric acid	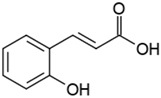	[41]
	Caffeic acid	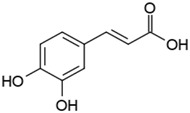	[42]
	Chlorogenic acid	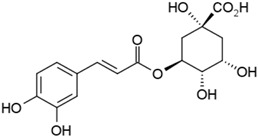	[42]
Lignans	Podophyllotoxin	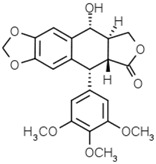	[43]
Curcuminoids	Curcumin	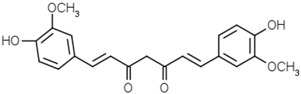	[44,45]
Flavonols	Quercetin	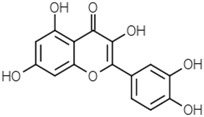	[23]
Isoflavonoids	Genistein	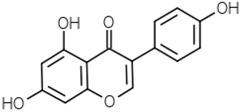	[46,47,48]
	Daidzein	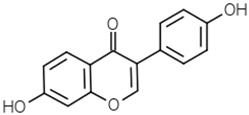	[47,48]
	Glycitein	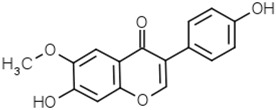	[48]
Flavones	Apigenin	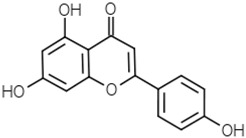	[49]
	Luteolin	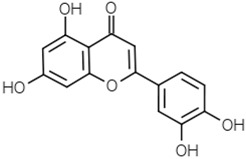	[50]
	Tangeritin	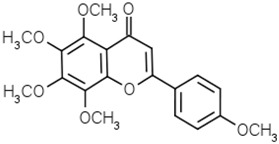	[51]
Flavans-3-ol	Catechin	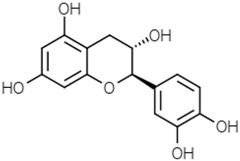	[39]
Anthocyanins	Cyanidin	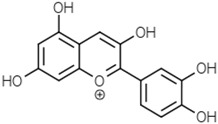	[52]
	Delphinidin	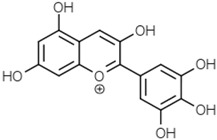	
	Malvidin	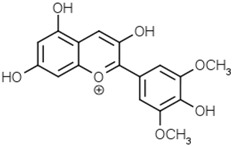	
	Pelargonidin	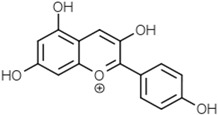	
	Peonidin	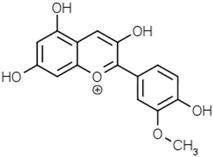	
Phytosterols	Diosgenin	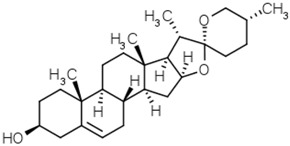	[53]
	Brassicasterol	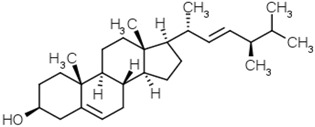	
	β-Sitosterol	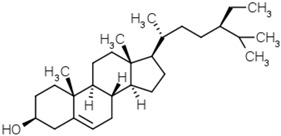	
	Campesterol	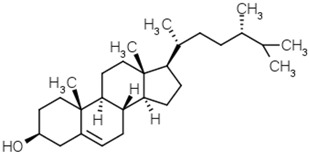	
Alkaloids	Caffeine	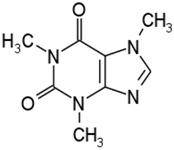	[53]
	Capsaicin	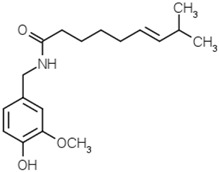	[54]
